# OP11 Assessing Patient And Societal Unmet Needs: The Needs Examination, Evaluation And Dissemination (NEED) Assessment Framework

**DOI:** 10.1017/S0266462324000758

**Published:** 2025-01-07

**Authors:** Charline Maertens de Noordhout, Muriel Levy, Claudia Schönborn, Mats De Jaeger, Laurence Kohn, Rani Claerman, Robby De Pauw, Irina Cleemput

## Abstract

**Introduction:**

The healthcare ecosystem is predominantly supply-driven, leading to a lack of innovation in financially less-attractive health areas, inefficient use of public resources for healthcare, and unmet patient and societal needs. This study proposes a framework to identify unmet health-related patient and societal needs for different health conditions, to inform and support the development of needs-driven healthcare policy and innovations.

**Methods:**

Two systematic literature reviews were conducted: one to update an already published literature review that identified tools for measuring patient needs; another to identify criteria for measuring health-related societal needs. Ovid MEDLINE and Embase were searched for English, French, and Dutch publications. In April 2023, 22 Belgian stakeholders and experts reviewed selected societal needs criteria. The NEED (Needs Examination, Evaluation and Dissemination) assessment framework, incorporating literature-derived patient and societal needs criteria, underwent discussions with the Health Minister’s Cabinet and federal health agencies from May to July 2023. The framework was challenged in November 2023 during international and national advisory committee meetings.

**Results:**

The literature reviews covered 52 studies. The NEED framework addresses patient, societal, and future needs across health, healthcare, and social domains. Patient-level criteria (13) include health (e.g., impact on quality of life), healthcare (e.g., burden of treatment), and social (e.g., social support). Societal needs criteria (6) encompass health (e.g., transmissibility), healthcare (e.g., value for money), and social (e.g., productivity losses). Future needs criteria (2) consider future burden of disease and economic burden. Each criterion (total: 40) has measurable indicators with calculation methods and data sources. Equity is recognized as a transversal dimension, requiring unmet needs data disaggregated by population group.

**Conclusions:**

For the first time, a transparent scientific framework containing criteria and indicators for assessing the unmet needs of patients and society has been developed. This lays the foundations for a major shift towards a more needs-driven healthcare policy and innovation.

